# Association of TPH-1 and TPH-2 gene polymorphisms with suicidal behavior: a systematic review and meta-analysis

**DOI:** 10.1186/1471-244X-14-196

**Published:** 2014-07-08

**Authors:** Thelma Beatriz González-Castro, Isela Juárez-Rojop, María Lilia López-Narváez, Carlos Alfonso Tovilla-Zárate

**Affiliations:** 1 División Académica de Ciencias de la Salud, Universidad Juárez Autónoma de Tabasco, Villahermosa, Tabasco, México; 2 CIGEN, Centro de Investigación Genómica, Comalcalco, Tabasco, México; 3 Hospital General de Yajalón, Yajalón, Chiapas, México; 4 División Multidisciplinaria de Comalcalco, Universidad Juárez Autónoma de Tabasco, Ranchería Sur, Cuarta Sección, Comalcalco, Tabasco, C.P. 86650, México

**Keywords:** Suicidal behavior, Systematic review, Meta-analysis, TPH-1 gene, TPH-2 gene

## Abstract

**Background:**

It is widely acknowledged that suicidal behavior (SB) has a genetic influence. As a consequence, molecular genetic studies have been mostly conducted on serotonergic genes. One of the most promising candidate genes of this system is tryptophan hydroxylase (TPH). Although there have been several positive studies associating TPH genes and SB, the evidence is not entirely consistent. Therefore, we performed a meta-analysis to gain a better understanding into this issue.

**Methods:**

The meta-analysis was conducted with 37 articles of genetic association studies of TPH-1 (A218C and A779C) and TPH2 (G-703 T, A-473 T and G19918A) genes. To analyze the association of these variants with SB we used the following models: allelic, additive, dominant and recessive. In addition, we performed a sub-group analysis by Caucasian and Asian populations using the same four models.

**Results:**

TPH-1 gene variants showed a positive significant association with SB, but only in the fixed effects models. With regard to TPH-2 gene variants we could not find an association with SB.

**Conclusions:**

The study provides evidence that A218C/A779C TPH-1 variants may be a risk factor to manifest SB at the clinical level, which is in agreement with previously reported meta-analyses. With regard to G-703 T/A-473 T/G19918A TPH-2 variants, our up-to-date meta-analysis could not detect any significant association between those genetic variants and SB. However, these results should be interpreted with caution since further studies need to be undertaken using larger sample sizes in different ethnic populations to confirm our findings.

## Background

Suicidal behavior (SB) is a leading cause of injury and death worldwide; it accounts for almost one million deaths annually and is the leading cause of death among individuals between 15 and 44 years of age [1-3]. Suicide has been defined as the act of intentionally ending one’s own life. Nonfatal suicidal thoughts and behaviors are classified more specifically into three categories: suicide ideation (refers to thoughts and plans for ending one’s life), suicide attempt (engagement in potentially self-injurious behavior with no lethal outcome) and completed suicide (the person ends with his/her life). There are several measurements of past suicidal behavior, which basically consist in predicting future suicidal behavior, as well as to obtain information about history ideation, plans and/or attempts to commit suicide, among other data (e.g. DSM-IV diagnoses) [4-6].

A genetic influence in SB is now widely acknowledged and has received convincing support from familial, twin and adoption studies [7,8], together with molecular genetic studies mostly conducted on serotonergic candidate genes. Moreover, the evidence from these various genetic studies indicates that dysfunctions of the central serotonergic system are involved in the pathogenesis of suicidal behavior [9]. In this manner, of the several serotonin-related genes studied in relation to SB, one of the most promising is tryptophan hydroxylase (TPH) [10]. The principal reason for testing the association between TPH genes and SB is that TPH is the first and rate-limiting step in the synthesis of the serotonin neurotransmitter [11]. TPH genes have been associated with low CSF 5-HIAA levels in healthy volunteers and have been suggested as a quantitative risk factor, in which a greater effect of the gene results in the intensification of serotonergic dysfunction associated with higher levels of anger and more severe suicidal acts [12,13].

With regard to the TPH enzyme there are two genes encoding it, both lie on intron seven: TPH-1, located on the human chromosome 11p15.3-14, has an approximate length of 29 kb and contains at least 11 exons and TPH-2 on chromosome 12q 21.1 covers a region of about 97 kb and comprises also 11 exons [14]. Thus, variations in TPH genes have prompted several researchers to conduct association genetic studies on TPH single-nucleotide polymorphisms (SNPs) [15]. Of the several different polymorphisms studied, most researches have focused on two SNPs in TPH1 [16]. The first polymorphism, A779C (rs1799913), was identified by single strand conformational analysis and comes mainly in two alternative forms or alleles TPH 779A and TPH 779C, referred to as U and L, respectively [17]. The second polymorphism, A218C (rs1800532), is in complete or strong linkage disequilibrium with A779C in various populations; both have been studied somewhat interchangeably [9]. With regard to TPH2 gene variants, the association with suicidal behavior is in progress and until now several SNPs have been studied [10]. However, the most common SNPS studied in connection with SB are rs4570625 (G-703 T), rs11178997 (A-473 T) and rs1386494 (G19918A) [18]. These SNPs have been selected for their functional relevance. As a result, the G-703 T polymorphism has been reported in higher number in suicidal depressed patients compared with healthy controls, whereas the A-473 T polymorphism has raised large interest for its possible effect on gene expression in the brainstem of depressed patients who committed suicide [19]. Finally, the G19918A polymorphism has been found in association with completed suicides [20].

Although there have been several positive studies associating TPH genes and SB the evidence is inconsistent. These inconclusive results are probably due to small sample sizes, different polymorphic markers used in the analysis, as well as variations in sampling strategies, mainly in the control groups, and the different ethnic populations assessed [21]. Although there are previous meta-analyses [18,22-25], we decided to undertake another study in which in larger number sample size and more rigorous selection criteria could give us a better understanding into this matter. The aims of the present study were to analyze the association of TPH-1 (A218C and A779C) and TPH2 (G-703 T, A-473 T and G19918A) genes with SB using a meta-analysis and to integrate the outcomes of the several genetic studies included in this methodology by taking into account heterogeneity sources and the evaluation of possible bias in publication. Given that several authors have shown that the frequencies of TPH-1 and TPH-2 gene variants may be ethnic-dependent, we decided to perform a sub-group analysis by populations.

### PICOT question

In population with suicidal behavior, what is the influence of TPH-1 and TPH2 genes variants in manifesting a clinical expression of suicidal behavior when compared to healthy subjects?

## Methods

The research protocol of this analysis is available upon request. The meta-analysis and systematic review were performed by following the Preferred Reporting Items for Systematic Reviews and Meta-Analyses (PRISMA) criteria [26,27].

### Identification and selection of publications

Data were obtained by searching in EBSCO and PubMed databases from May to Jun 2013. Relevant publications were identified using the following search terms: “TPH1 and suicidal behavior” (PubMed: 23; EBSCO: 17), “TPH2 and suicidal behavior” (PubMed: 28; EBSCO: 29), “TPH1 and suicide” (PubMed: 25; EBSCO: 17), “TPH2 and suicide” (PubMed: 46; EBSCO: 39), “Tryptophan hydroxylase and suicidal behavior” (PubMed: 90; EBSCO: 53). These words were combined to retrieve the summaries. The search also implicated the review of the bibliography cited at the end of the various research articles. To be selected, the publications had to meet the following criteria: (1) to be published in peer-reviewed journals, (2) to contain independent data, (3) to be case–control association studies in which the frequencies of three genotypes were clearly stated or could be calculated, (4) diagnosis of suicidal behavior of the patients in the study (suicide ideation, suicide attempt and completed suicide), and (5) the use of healthy individuals as controls. A final requirement was that the articles were written in English. The following polymorphisms were studied: A779C (rs1799913) and A218C (rs1800532) of the TPH-1 gene, and rs4570625 (G-703 T), rs11178997 (A-473 T), and rs1386494 (G19918A) of the TPH-2 gene.

Two researchers (González-Castro and Juárez-Rojop) working independently screened each of the titles, abstracts, and full texts to determine inclusion. When the researchers were in disagreement a third researcher (Tovilla-Zárate) was consulted.

### Data extraction

The same authors mentioned above carefully extracted the information from all the included publications. They worked in an independent manner and in accordance with the inclusion criteria listed above. The following data were obtained from each of the studies: authors, year of publication, region, number of cases and controls, age, gender, psychiatric diagnosis, number of males in the groups and ethnical origin of the participants. These data were not always available for all the studies. In the cases of missing data, we contacted the respective authors to ask for the allele frequencies not included in the main text of the papers. One of the studies did not include a control group [28], but we made an adjustment accordingly based on the other studies in the literature that included a control group [29]. Briefly, we calculated the weighted frequency for a particular genotype from studies that included controls and applied it to the study without a control group. We considered the number of the “virtual” control group equal to the number of patients in the specific study. Then the hypothetical number of subjects with the particular genotype frequency was assigned in proportion to the percentage of the same genotype which was obtained from the weighted analysis [30,31]. The outcomes of the meta-analysis were built by taking into consideration the categories reported in previously published studies [32,33].

**Figure 1:**
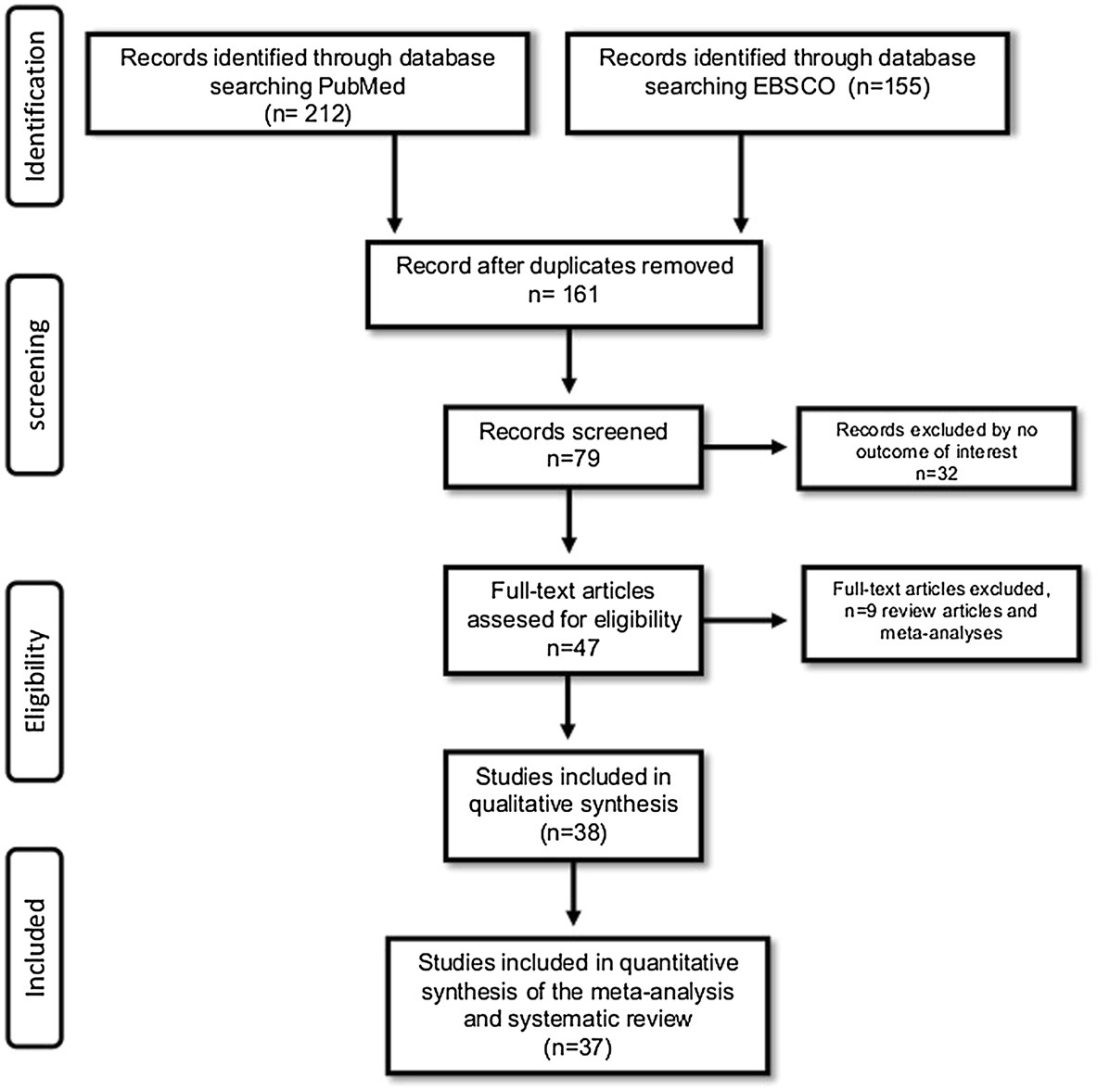
Flow-chart showing the search strategy and inclusion/exclusion criteria used in the meta-analysis and systematic review.

**Figure 2:**
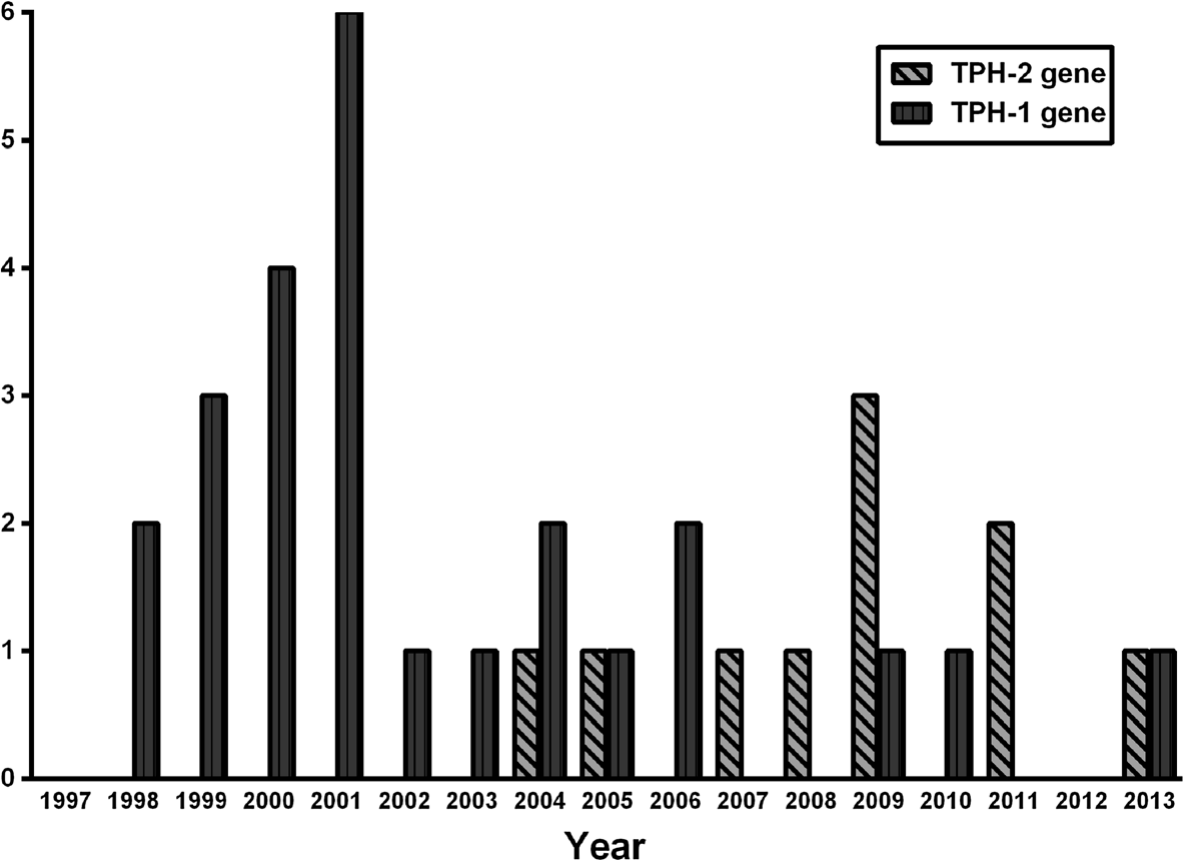
Distribution of association studies (cases-controls) on TPH genes variants and SB published in PubMed by year.

Figure 1 shows the stages of the meta-analysis. The combined search included 367 potentially relevant articles. Finally, 37 articles were obtained after discarding overlapping references that did not agree with the inclusion criteria [16,17,19-24,28,34-61]; Figure 2 shows the articles published at this stage of the analysis. In the case of TPH-1 gene variants (A218C and A779C), 5683 cases and 11652 controls were involved, whereas for the TPH-2 gene variants (G-703 T, A-473 T and G19918A) 4196 cases and 5990 controls were considered (Tables 1 and 2, respectively).

**Table 1:** Descriptive characteristics in association studies on A218C/A779C TPH-1 gene variants and SB by populations

**Reference**	**Location**	**Diagnosis**	**Number of males**		**Number**		**Genotypes**		**Alleles**		**P for HWE**	
			**Cases**	**Controls**	**Cases**	**Controls**	**Cases**	**Controls**	**Cases**	**Controls**	**Cases**	**Controls**
							**AA/AC/CC**	**AA/AC/CC**	**A/C**	**A/C**		
***Caucasian population (A218C TPH-1 variant)***												
Bellivier F. et al., 1998 [62]	France	SA and manic-depression	-	57	104	94	34/48/22	11/45/38	116/92	67/121	0.08	0.55
Geijer T. et al., 2000 [53]	Sweden	SA	57	64	165	98	30/87/48	13/47/38	147/183	73/123	1.00	0.43
Du L. et al., 2000 [54]	Canada	SC	-	-	35	84	6/24/5	13/52/19	36/34	78/90	0.04*	0.04*
Souery D. et al., 2001 [37]	Germany, Greece, Israel, Italy, Sweden, UK.	SI and bipolar disorder	-	-	167	167	29/85/53	27/74/66	143/191	128/206	0.41	0.75
Abbar M. et al., 2001 [47]	France	SA	105	170	231	281	43/120/68	30/133/118	206/256	193/369	0.50	0.30
Zalsman G.et al., 2001 [40]	Israel	SA	40	74	84	172	16/34/34	41/85/46	66/102	167/178	0.89	0.17
Turecki G. et al., 2001 [39]	Canada	SC	-	-	101	129	18/48/35	18/71/40	84/118	107/151	0.15	0.83
Rujescu D. et al., 2002 [36]	Germany	SA and anger related traits	27	66	86	154	10/48/28	19/78/57	68/104	116/192	0.39	0.17
Rujescu D. et al., 2003 [24]	Germany	SA	52	-	147	326	18/81/48	40/155/131	117/177	235/417	0.63	0.08
Jernej B. et al., 2004 [34]	Croatia	SC	144	298	185	358	84/85/16	202/141/15	253/117	545/171	0.14	0.49
Stefulj J. et al., 2005 [16]	Croatia	SC	160	284	160	284	21/69/70	44/145/95	111/209	233/335	0.39	0.60
Stefulj J. et al., 2006 [46]	Croatia	SC	247	320	247	320	33/111/103	50/162/108	177/317	262/378	0.48	0.78
Viana M.M. et al., 2006 [48]	Brazil	SA, major depression, schizophrenia and alcoholism	142	36	248	63	45/125/78	7/31/25	215/281	45/81	0.78	0.79
Baud P. et al., 2009 [28]	Switzerland, France	SA	166	-	537	1027	99/256/182	181/483/363	454/260	845/1209	0.59	0.34
Wilson T.et al., 2009 [52]	USA	SA and bipolar disorder	-	39	71	101	13/44/14	22/43/36	70/72	87/115	0.22	0.05*
Saetre P. et al., 2010 [23]	Denmark, Norway, Sweden	SA and schizophrenia	-	845	825	1460	289/391/145	570/694/196	969/681	1834/1086	0.00*	0.63
Buttenschon H.N. et al., 2013 [41]	Denmark	SC	209	763	490	1027	80/228/182	181/843/363	388/592	845/1209	0.36	0.57
							868/1884/1131	1469/3282/1754			0.11	0.37
***Asian population (A218C TPH-1 variant)***												
Tsai S.J. et al., 1999 [55]	Taiwan	SA and mood disorders	-	-	41	200	17/15/9	33/113/54	49/33	179/221	0.06	0.19
Liu X. et al., 2006 [56]	China	SA	171	98	287	177	72/148/67	41/84/49	292/282	166/182	0.65	0.63
Yoon H.K. et al., 2008 [44]	Korea	SA and major depression	70	73	193	191	49/97/45	60/85/48	195/187	205/181	0.11	0.88
Kunugi H. et al.,1999 [42]	Japan	SA	-	95	46	208	10/29/7	55/105/48	49/43	215/201	1.00	0.13
Paik I. et al., 2000 [45]	Korea	SA and schizophrenia	-	124	27	236	04/12/11	66/116/54	20/34	248/224	0.89	1.00
Ono H., et al., 2000 [43]	Japan	SC	90	90	132	132	29/68/35	26/71/35	126/138	123/141	0.38	0.86
Hong C.J. et al., 2001 [35]	Taiwan	SA and schizophrenia	-	123	140	251	42/57/41	42/135/74	141/139	219/283	0.15	0.02*
Ohtani M. et al., 2004 [49]	Japan	SC	95	171	134	325	30/60/42	44/113/57	120/144	201/227	0.41	0.37
							253/486/257	367/822/419				
***Caucasian population (A779C TPH-1 variant)***												
Nielsen D.A. et al.,1998 [38]	USA	SI and alcoholism	-	232	102	232	17/57/28	49/106/77	91/113	204/260	0.28	0.23
Rotondo A. et al., 1999 [51]	Italy and USA	SA	-	153	97	153	12/50/35	33/68/52	74/120	215/201	0.25	0.51
Roy A. et al., 2001 [17]	Sweden	SC	-	-	24	158	02/12/20	35/86/37	16/332	156/160	0.33	1.00
Saetre P. et al., 2010 [23]	Denmark, Norway and Sweden	SA and schizophrenia	-	845	830	1464	290/391/149	570/691/203	971/689	1831/1097	0.00*	0.63
Pooley E.C. et al., 2003 [50]	UK	SA	52	138	129	329	20/67/42	44/135/150	107/151	223/435	0.13	0.47
							341/577/274	731/1086/519			0.44	0.34
***Asian population (A779C TPH-1 variant)***												
Kunugi H. et al., 1999 [42]	Japan	SA	-	95	46	208	10/29/7	55/105/48	49/43	215/201	1.00	0.13
Ohtani M. et al., 2004 [49]	Japan	SC	95	171	134	325	30/59/42	45/138/82	119/143	228/302	0.38	0.29
Liu X., et al. 2006 [56]	China	SA	171	98	266	164	77/126/63	43/85/36	280/252	171/157	0.75	0.46
							117/214/112	143/328/166			0.50	0.47

SA = Suicide attempt, SI = Suicide ideation, and SC = Suicide completion.* Non equilibrium of Hardy Weinberg.

**Table 2:** Descriptive characteristics in association studies on G-703 T/A473T/G19918A TPH-2 gene variants and SB by populations

**Reference**	**Location**	**Diagnosis**	**Number of males**		**Number**		**Genotypes**		**Alleles**		**P for HWE**	
			**Cases**	**Controls**	**Cases**	**Controls**	**Cases**	**Controls**	**Cases**	**Controls**	**Cases**	**Controls**
							**GG/TG/TT**	**GG/TG/TT**	**T/G**	**T/G**		
***Caucasian population (G-703 T TPH-2 variant)***												
Zhou Z. et al., 2005 [61]	USA	SA and major depression	150	196	150	196	106/40/4	132/58/6	252/48	322/70	1.00	1.00
Zill P. et al., 2007 [60]	Germany	SA and alcoholism	-	164	102	305	61/36/5	191/101/13	158/46	483/127	1.00	1.00
Stefulj J. et al., 2011 [57]	Croatia	SC	291	280	291	280	181/96/14	183/80/17	458/124	446/114	0.06	0.72
							348/172/23	506/239/36			0.78	0.26
***Asian population (G-703 T TPH-2 variant)***												
Mouri K. et al., 2009 [58]	Japan	SC	156	162	234	260	62/117/55	80/128/52	241/227	288/232	1.00	1.00
Yoon H.K. et al., 2008 [44]	Korea	SA and major depression	82	80	181	176	58/81/42	34/82/60	197/165	150/202	0.18	0.53
							120/198/97	114/210/112			0.37	0.44
**Reference**	**Location**	**Diagnosis**	**Number of males**		**Number**		**Genotypes**		**Alleles**		**P for HWE**	
			**Cases**	**Cases**	**Cases**	**Controls**	**Cases**	**Controls**	**Cases**	**Controls**	**Cases**	**Controls**
							**AA/AT/TT**	**AA/AT/TT**	**A/T**	**A/T**		
***Caucasian population (A-473 T TPH-2 variant)***												
Zhou Z. et al., 2005 [61]	USA	SA and major depression	150	196	150	196	0/14/136	0/15/181	14/286	15/377	1.00	1.00
Zupanc T.et al., 2011 [19]	Slovenia	SC and alcoholism	-	-	383	222	0/42/341	2/31/189	42/724	35/409	0.61	0.63
							0/56/177	2/46/370			0.36	0.65
***Asian population (A-473 T TPH-2 variant)***												
Mouri K.et al., 2009 [58]	Japan	SC	156	162	234	260	2/44/188	3/48/209	48/420	54/466	1.00	0.74
**Reference**	**Location**	**Diagnosis**	**Number of males**		**Number**		**Genotypes**		**Alleles**		**P for HWE**	
			**Cases**	**Cases**	**Cases**	**Controls**	**Cases**	**Controls**	**Cases**	**Controls**	**Cases**	**Controls**
							**GG/GA/AA**	**GG/GA/AA**	**G/A**	**G/A**		
***Caucasian population ( G19918A TPH-2 variant)***												
Zill P. et al.,2004 [20]	Germany	SC	191	138	263	266	195/63/5	166/88/12	453/73	420/112	1.00	1.00
Zill P. et al.,2007 [60]	Germany	SA and alcoholism	-	164	102	305	4/31/67	12/98/195	39/165	122/488	0.75	1.00
Must A. et al., 2009 [59]	Estonia	SC	298	327	288	327	206/75/7	242/79/6	487/89	563/91	1.00	1.00
Buttenschon H.N. et al.,2013 [41]	Denmark	SC	209	763	553	1033	396/150/7	743/268/22	942/169	1754/312	0.09	0.08
							801/319/86	1163/533/235			0.06	0.09
***Asian population ( G19918A TPH-2 variant)***												
K. Mouri et al.2009 [58]	Japan	SC	156	162	234	260	219/15/0	239/21/0	453/234	499/260	1.00	1.00

SA = Suicide attempt, SI = Suicide ideation, and SC = Suicide completion.

### Data analysis

For the meta-analysis procedures we used the EPIDAT 3.1 program (http://www.sergas.es/MostrarContidos_N3_T01.aspx?IdPaxina=62715). This software is freely available for epidemiologic analysis of tabulated data. Data were analyzed with the random-effects and fixed effects models following the reports in the literature [63-65]. Sample heterogeneity was analyzed with the Dersimonian and Laird’s Q test. Q test results were complemented with graphs to help the visualization of those studies favoring heterogeneity. The studies outside the heterogeneity curve were excluded. The results of the meta-analysis are expressed as odds ratios (ORs). Pooled ORs were calculated respectively for each of the models used, viz.: allelic (Example: A vs. C), additive (AA vs. CC), dominant (AA + AC vs. CC), and recessive (AA vs. AC + CC). Also, we analyzed TPH1 and TPH2 gene variants by Caucasian and Asian populations. To address the problem of publication bias, the Egger’s test and funnel plots were calculated with the EPIDAT 3.1 software. This plotting standardizes the effect of each of the published studies on the vertical axis and its correspondent precision on the horizontal axis. Also, we evaluated publication bias using the GRADE approach and assessed the risk for bias. Finally, a chi-squared (χ^2^) analysis was used to calculate the Hardy-Weinberg equilibrium to evaluate genotype distribution. Studies deemed for inclusion in the systematic review were scored for methodological quality using the Newcastle-Ottawa Assessment Scale (NOS). A score of six or more was taken as the cut-off point to distinguish high from low quality studies [66] (see Additional file 1).

## Results

We measured the Hardy-Weinberg equilibrium (HWE) in both groups (cases and controls) for all the samples of TPH genes; we also explored all populations in a combined way. Results are shown in Tables 1 and 2.

### TPH-1 gene variants

#### A218C polymorphism

**Figure 3:**
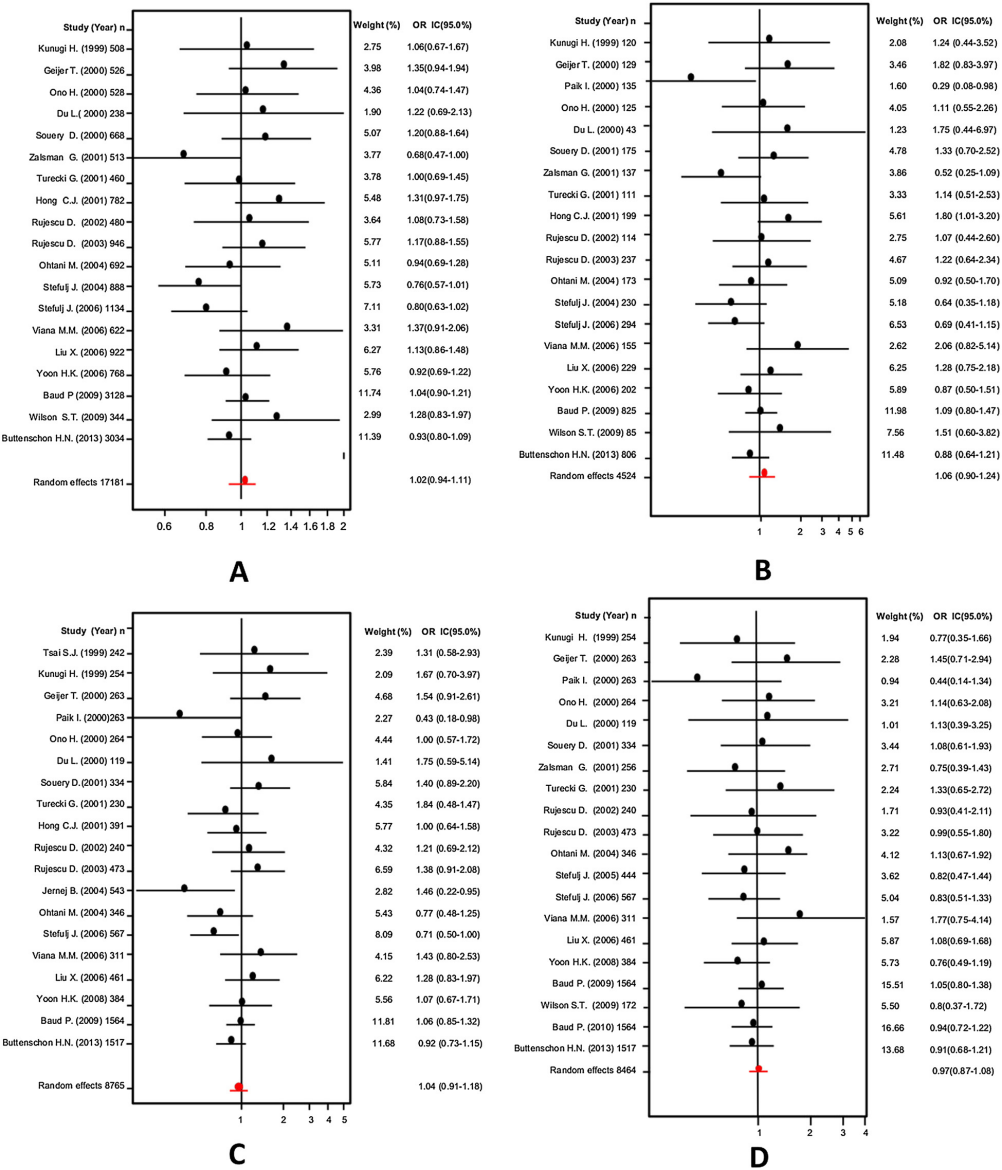
Odds ratios and forest plots of A218C in overall studies without heterogeneity, using the following models: A) Allelic; B) Additive; C) Dominant, and D) Recessive.

**Figure 4:**
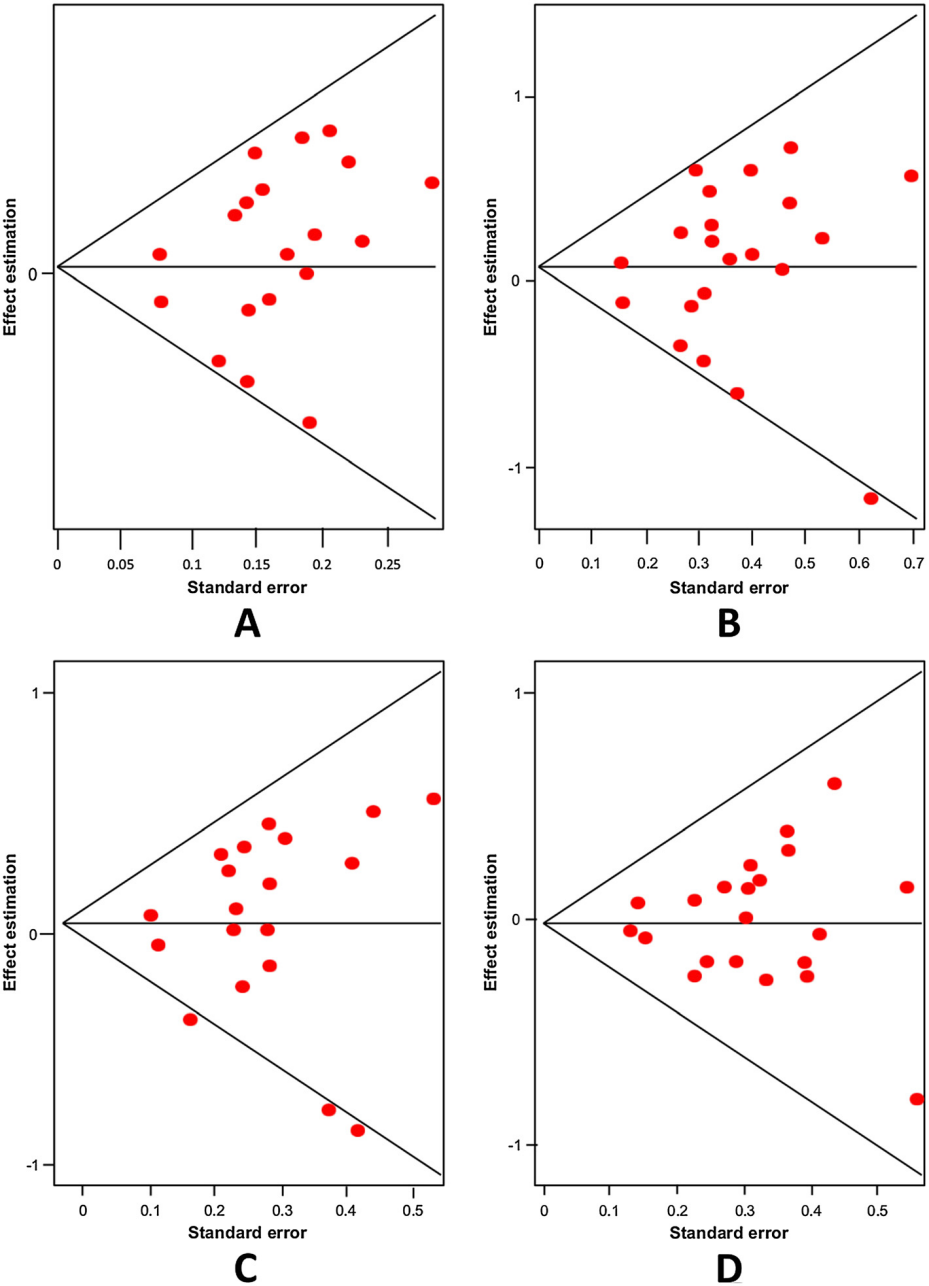
Egger’s funnel plots indicating publication bias in studies on suicidal behavior and the A218C variant without heterogeneity, using the following models: A) Allelic; B) Additive; C) Dominant, and D) Recessive models.

Firstly, an analysis of the variant A218C (rs1800532) of the TPH-1 gene was performed. In this study we evaluated the role of this variant in several models: allelic, additive, dominant and recessive. Also, we considered the Caucasian and Asian populations separately, using the same four models. And as in a previous meta-analysis [18,22-25], we found a positive association of A218C variant with SB in the fixed effects model, but not in the random effects model. Our results are presented in Table 3. Figures 3 and 4 present the forest plots and Egger’s test of the same four models previously mentioned (allelic, additive, dominant and recessive).

**Table 3:** Meta-analysis of case–control studies on A218C/A779C TPH-1 gene variants in patients with suicidal behavior

**Model analysis**		**A218C**		**P value of Q test**	**P value of Egger’s test**	**A779C**		**P value of Q test**	**P value of Egger’s test**
		**Random effects**	**Fixed effects**			**Random effects**	**Fixed effects**		
		**OR (CI 95%)**	**OR (CI 95%)**			**OR (CI 95%)**	**OR (CI 95%)**		
**All populations**									
**Allelic**	With heterogeneity	1.10 (0.98-1.22)	**1.09 (1.04-1.16)**	<0.001	0.85	0.96 (0.76-1.22)	1.09 (0.99-1.21)	<0.0001	0.46
	Without heterogeneity	1.02 (0.94-1.11)	1.01 (0.95-1.08)	0.11	0.27	0.97 (0.85-1.11)	0.97 (0.85-1.11)	0.43	0.24
**Additive**	With heterogeneity	1.22 (0.98-1.52)	**1.22 (1.09-1.37)**	<0.00001	0.95	1.02 (0.67-1.54)	**1.29 (1.04-1.59)**	0.001	0.25
	Without heterogeneity	1.06 (0.90-1.24)	1.04 (0.92-1.19)	0.15	0.50	0.92 (0.71-1.19)	0.92 (0.71-1.19)	0.42	0.11
**Dominant**	With heterogeneity	1.16 (0.96-1.41)	**1.16 (1.07-1.27)**	<0.0001	0.93	1.17 (0.75-1.83)	**1.43 (1.21-1.69)**	<0.0001	0.07
	Without heterogeneity	1.04 (0.91-1.18)	1.02 (0.93-1.13)	0.07	0.65	1.00 (0.82-1.22)	1.00 (0.82-1.22)	0.47	0.73
**Recessive**	With heterogeneity	1.10 (0.94-1.28)	1.04 (0.95-1.15)	0.0005	0.15				
	Without heterogeneity	0.97 (0.87-1.08)	0.97 (0.87-1.08)	0.97	0.80	0.88 (0.72-1.08)	0.90 (0.76-1.06)	0.23	0.23
**Caucasian population**									
**Allelic**	With heterogeneity	1.12 (0.971.29)	1.10 (1.04-1.17)	<0.0001	0.69	0.90(0.62-1.30)	1.11(0.98 1.25)	<0.0001	0.60
	Without heterogeneity	1.01(0.91-1.13)	1.00 (0.93-1.08)	0.006	0.36	0.95(0.78-1.14)	0.95(0.78 1.14)	0.58	0.25
**Additive**	With heterogeneity	1.25 (0.95-1.64)	1.22 (1.07-1.39)	<0.0001	0.73	0.89(0.46-1.71)	1.36 (1.05 1.75)	<0.0001	0.80
	Without heterogeneity	1.05(0.87-1.27)	1.03 (0.88-1.19)	0.18	0.22	0.80(0.55-1.16)	0.80(0.55-1.16)	0.69	0.94
**Dominant**	With heterogeneity	1.24(0.96-1.59)	1.20 (1.09-1.32)	<0.0001	0.68	1.17(0.75-1.83)	1.43(1.21-1.69)	<0.0001	0.40
	Without heterogeneity	1.12(0.97-1.28)	1.10 (0.97-1.25)	0.35	0.06	1.09(0.83-1.44)	1.09(0.83 -1.44)	0.77	0.58
**Recessive**	With heterogeneity	1.02 (0.841.24)	0.97 (0.87-1.09)	<0.0001	0.34	0.81(0.67-0.98)	0.81(0.670.98)	<0.0001	0.30
	Without heterogeneity	0.95 (0.84-1.06)	0.95 (0.84-1.06)	0.66	0.20	0.68(0.51-0.91)	68(0.51-0.91)	0.71	0.31
**Asian population**									
**Allelic**	With heterogeneity	1.06(0.89-1.27)	1.06 (0.94-1.20)	0.04	0.72	1.05(0.88 1.27)	1.05(0.88-1.27)	0.93	0.84
	Without heterogeneity	1.11(0.95-1.28)	1.10 (0.97 1.24)	0.21	0.30				
**Additive**	With heterogeneity								
	Without heterogeneity	1.17(0.83-1.65)	1.18 (0.93 1.50)	0.06	0.69	1.15(0.79-1.68)	1.15(0.79-1.68)	0.83	0.76
**Dominant**	With heterogeneity					0.99(0.73-1.35)	0.99(0.73-1.35)	0.45	0.10
	Without heterogeneity	1.01(0.82-1.25)	1.01(0.84-1.23)	0.31	0.27	0.92(0.67.1.28)	0.92(0.67-1.28)	0.88	1.000
**Recessive**	With heterogeneity	1.18(0.81-1.72)	1.19(0.97-1.45)	0.002	0.89	1.16(0.86-1.58)	1.16(0.86-1.58)	0.40	0.54
	Without heterogeneity	0.94(0.74-1.18)	0.94 (0.74-1.18)	0.52	0.27				

Bold text: Statistically significant.

#### A779C polymorphism

**Figure 5:**
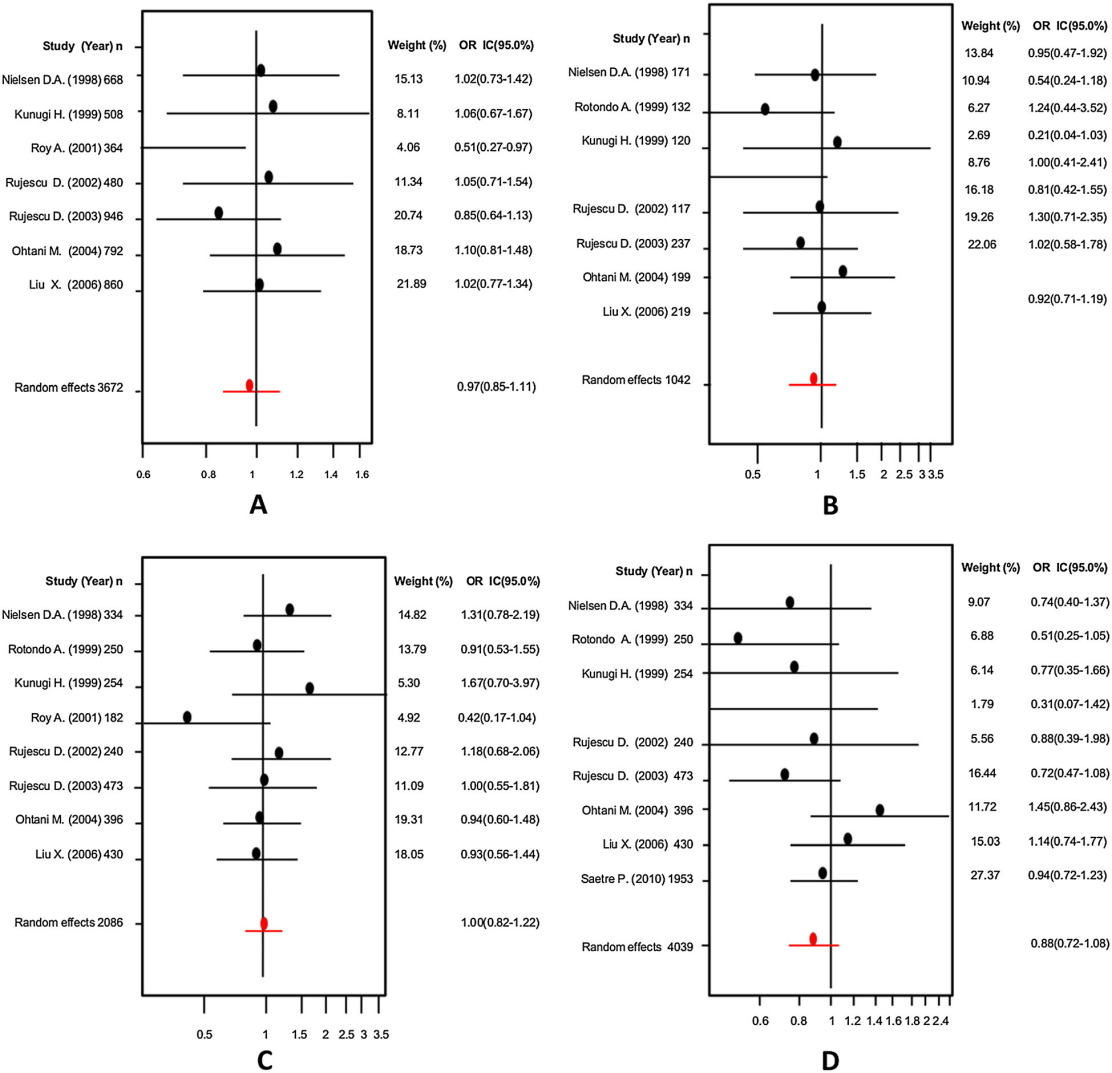
Odds ratios and forest plots of A779C in overall studies without heterogeneity, using the following models: A) Allelic; B) Additive; C) Dominant, and D) Recessive.

**Figure 6:**
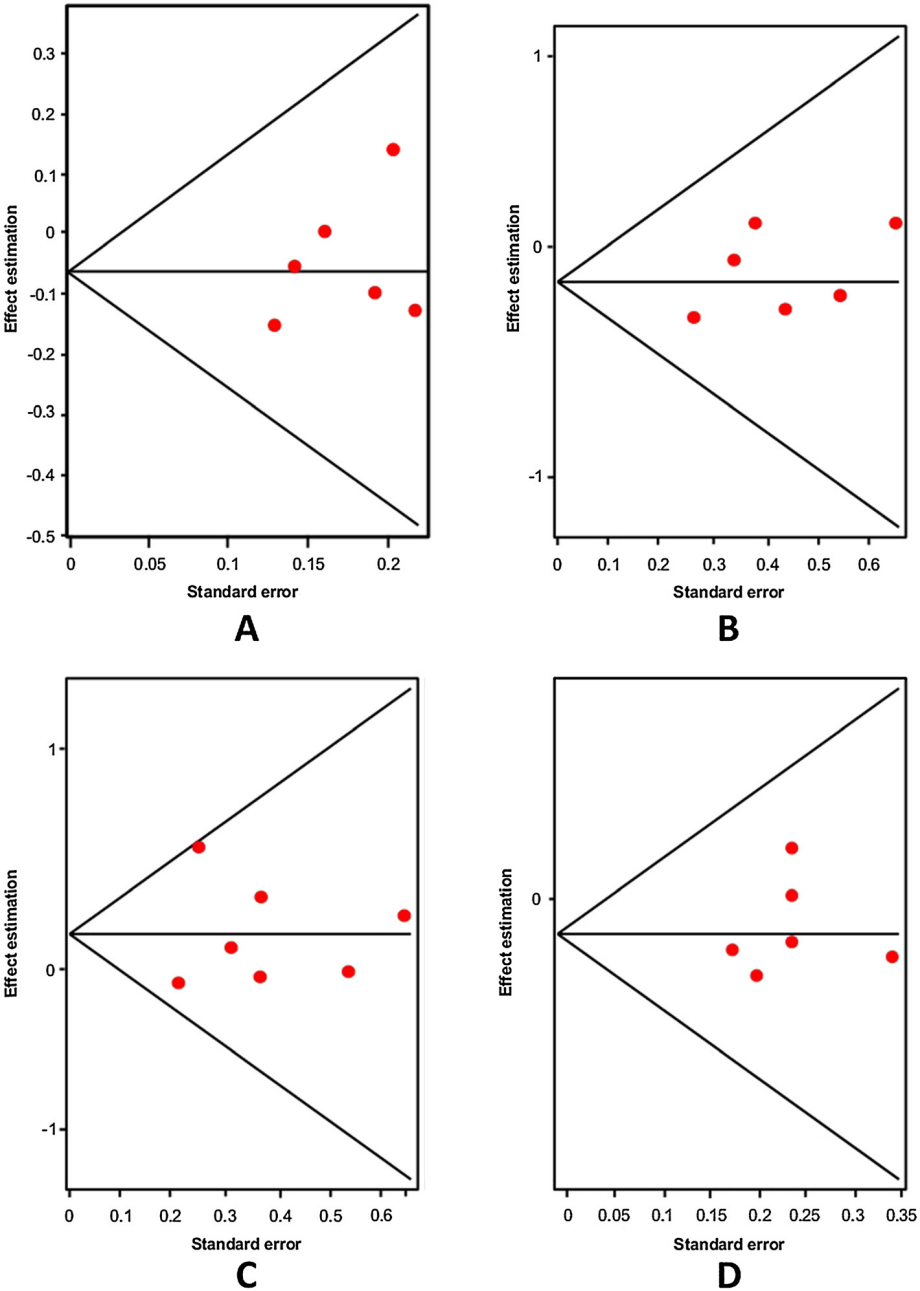
Egger’s funnel plots indicating publications bias in studies on suicidal behavior and the A779C variant without heterogeneity, using the following models: A) Allelic; B) Additive; C) Dominant, and D) Recessive models.

With this variant we conducted the same analyses than in the previous variant gene (A218C). We observed a significant association with suicidal behavior, using the fixed effects model. This analysis was extended to Caucasian and Asian populations treated separately. Table 3 shows in more detail the analysis of this variant gene in all the models tested (allelic, additive, recessive and dominant). Figures 5 and 6 present forest plots and Egger’s tests in the same models.

### TPH-2 gene variants

#### G703T polymorphism

**Figure 7:**
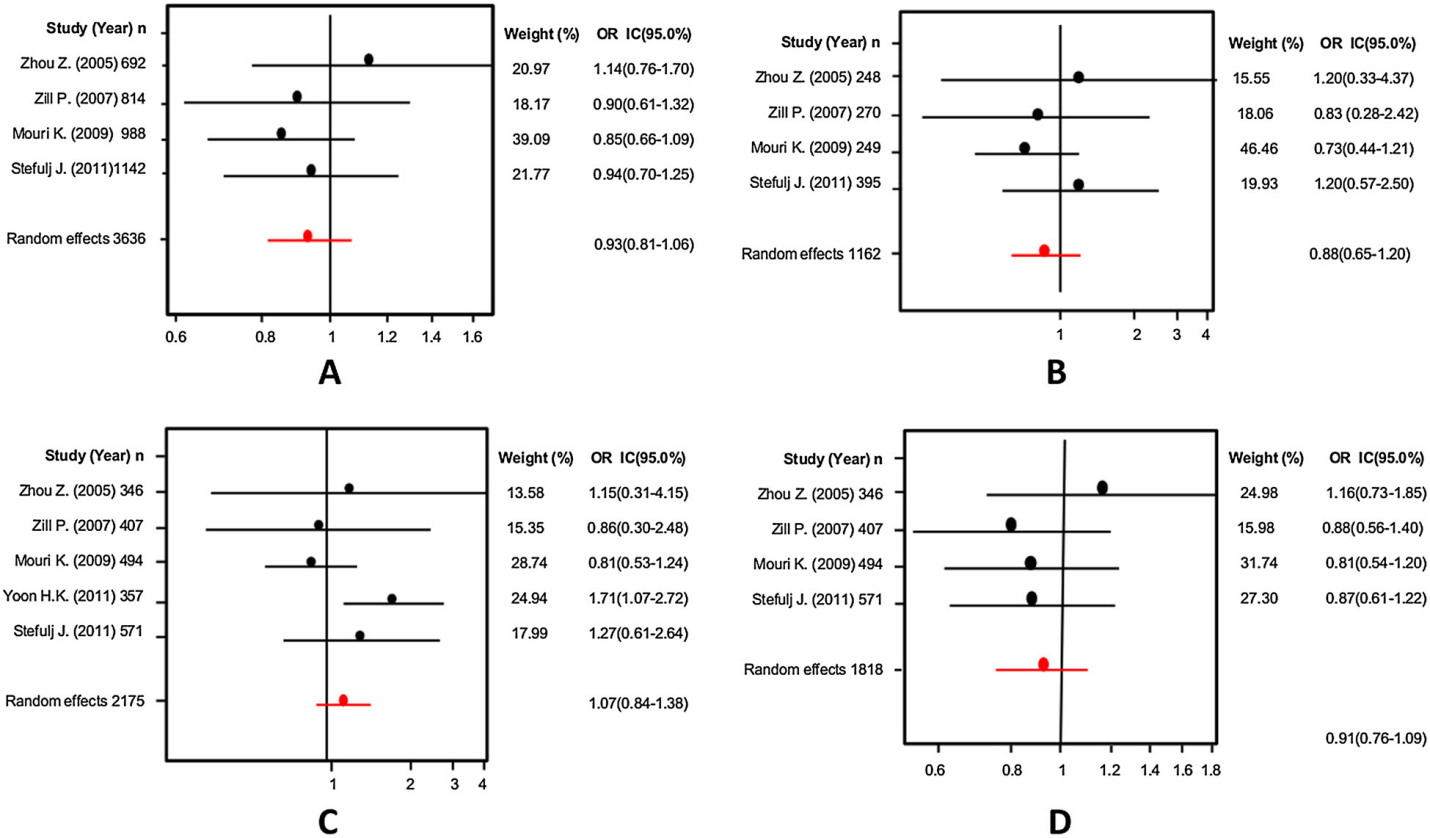
Odds ratios and forest plots of the G-703 T TPH-2 variant in overall studies without heterogeneity, using the following models: A) Allelic; B) Additive; C) Dominant, and D) Recessive.

**Figure 8:**
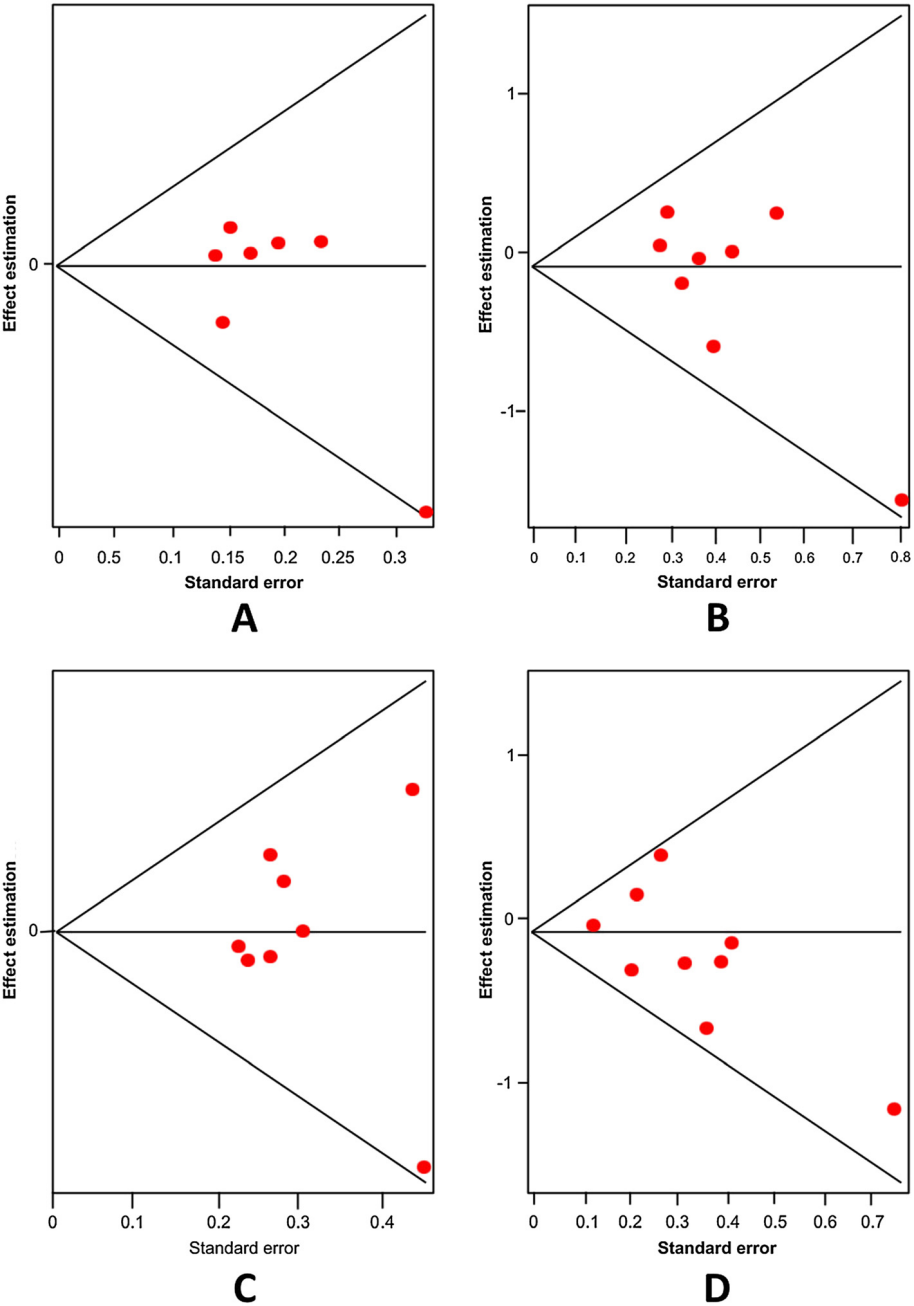
Egger's funnel plots indicating publication bias in studies on suicidal behavior and the G-703 T TPH-2 variant without heterogeneity, using the following models: A) Allelic; B) Additive; C) Dominant, and D) Recessive.

The first variant analyzed was the G-703 T (rs4570625) polymorphism. Initially, we explored the association of the G allele and patients with SB; we did not find significant differences (Random effects: OR: 1.02; 95% CI 0.85-1.22; p(Z) =0.99). We observed heterogeneity in all studies (Q = 12.43; df = 6; p = 0.04). The Egger's test provided no evidence of publication bias (t = 0.04; df = 5; p = 0.96) (see Additional file 1). Next, we excluded the study by Yoon and Kim [44], based on heterogeneity, and still we could not find any significant differences (Random effects: OR: 0.93; 95% CI 0.81-1.06; p(Z) =0.70; Figure 7A; Q test = 1.73; df = 5; p = 0.88). Also, there was no evidence of publication bias (Egger’s test: t = 1.01, df = 4; p = 0.36; Figure 8A). The same results were found when we used the additive model without heterogeneity (Random effects: OR: 0.88; 95% CI 0.65-1.20; p(Z) =0.45; Figure 7B; Q test = 1.65; df = 6; p = 0.89). No evidence of publication bias was encountered (Egger’s test t = 1.03; df = 5; p = 0.36; Figure 8B). We did not find any significant differences when we used the dominant model without heterogeneity either (Random effects: OR: 1.07; 95% CI 0.84-1.38; p(Z) =0.76; Figure 7C; Q test = 6.43; df = 6; p = 0.37). Similarly, no evidence of publication bias was observed (Egger’s test: t = 0.24, df = 5; p = 0.83; Figure 8). Finally, the same negative results were found in the recessive model without heterogeneity (Random effects: OR: 0.91; 95% CI 0.76-1.09; p(Z) =0.45; Q test = 1.76; df = 6; p = 0.88; Figure 7D). No evidence of publication bias was encountered (Egger’s test: t = 0.46; df = 5; p = 0.66; Figure 8D). In addition, we conducted the same analyses using the allelic, additive, dominant and recessive models in Asian and Caucasian populations, separately. However, we could not find any significant differences (Table 4).

**Table 4:** Meta-analysis of case–control studies on G-703 T/ A-473 T/G19918A TPH-2 gene variants and SB by populations

**Model analysis**		**G703T**		**P value of Q test**	**P value of Egger’s test**	**A473T**		**P value of Q test**	**P value of Egger’s test**	**G19918A**			
		**Random effects**	**Fixed effects**			**Random effects**	**Fixed effects**			**Random effects**	**Fixed effects**	**P value of Q test**	**P value of Egger’s test**
		**OR (CI 95%)**	**OR (CI 95%)**			**OR (CI 95%)**			**OR (CI 95%)**			**OR (CI 95%)**	**OR (CI 95%)**		
**Caucasian population**													
**Allelic**	With heterogeneity	0.95(0.79-1.14)	0.95(0.79-1.14)	0.91	0.72	0.95(0.79-1.14)	0.95(0.79-1.14)	0.91	0.72	1.12(0.88-1.43)	1.09(0.95-1.25)	0.04	0.59
	Without heterogeneity									0.99(0.85-1.16)	0.99(0.851.16)	0.52	0.57
**Additive**	With heterogeneity	1.03(0.66-1.62)	1.03(0.6-61.62)	0.84	0.56	1.03(0.66-1.62)	1.03(0.66-1.62)	0.84	0.56	1.45(0.87-2.40)	1.45(0.87-2.40)	0.44	0.95
	Without heterogeneity												
**Dominant**	With heterogeneity	1.05(0.68-1.62)	1.05(0.68-1.62)	0.79	0.79	1.05(0.68-1.62)	1.05(0.68-1.62)	0.79	0.79	1.18(0.79-1.76)	1.14(0.80-1.64)	0.35	0.33
	Without heterogeneity												
**Recessive**	With heterogeneity	0.91(0.72-1.15)	0.91(0.72-1.15)	0.86	0.54	0.91(0.72-1.15)	0.91(0.72-1.15)	0.86	0.54	1.15(0.86-1.55)	1.09(0.93-1.29)	0.06	0.59
	Without heterogeneity									0.98(0.81-1.18)	0.98(0.81-1.18)	0.60	0.52
**Asian population**													
**Allelic**	With heterogeneity	1.08(0.78-1.50)	1.08(0.92-1.27)	0.01	0.93								
	Without heterogeneity	0.91(0.75-1.11)	0.91(0.75-1.11)	0.47	0.58								
**Additive**	With heterogeneity	1.14(0.57-2.27)	1.14(0.81-1.60)	0.01	0.99								
	Without heterogeneity	0.78(0.51-1.17)	0.78(0.511.17)	0.78	0.29								
**Dominant**	With heterogeneity	1.09(0.82-1.44)	1.09(0.82-1.44)	0.11	0.94								
	Without heterogeneity												
**Recessive**	With heterogeneity	1.13 (0.74-1.70)	1.11(0.87-1.41)	0.03	0.86								
	Without heterogeneity	0.93(0.70-1.22)	0.93(0.70-1.22)	0.48	0.94								

#### A473T polymorphism

**Figure 9:**
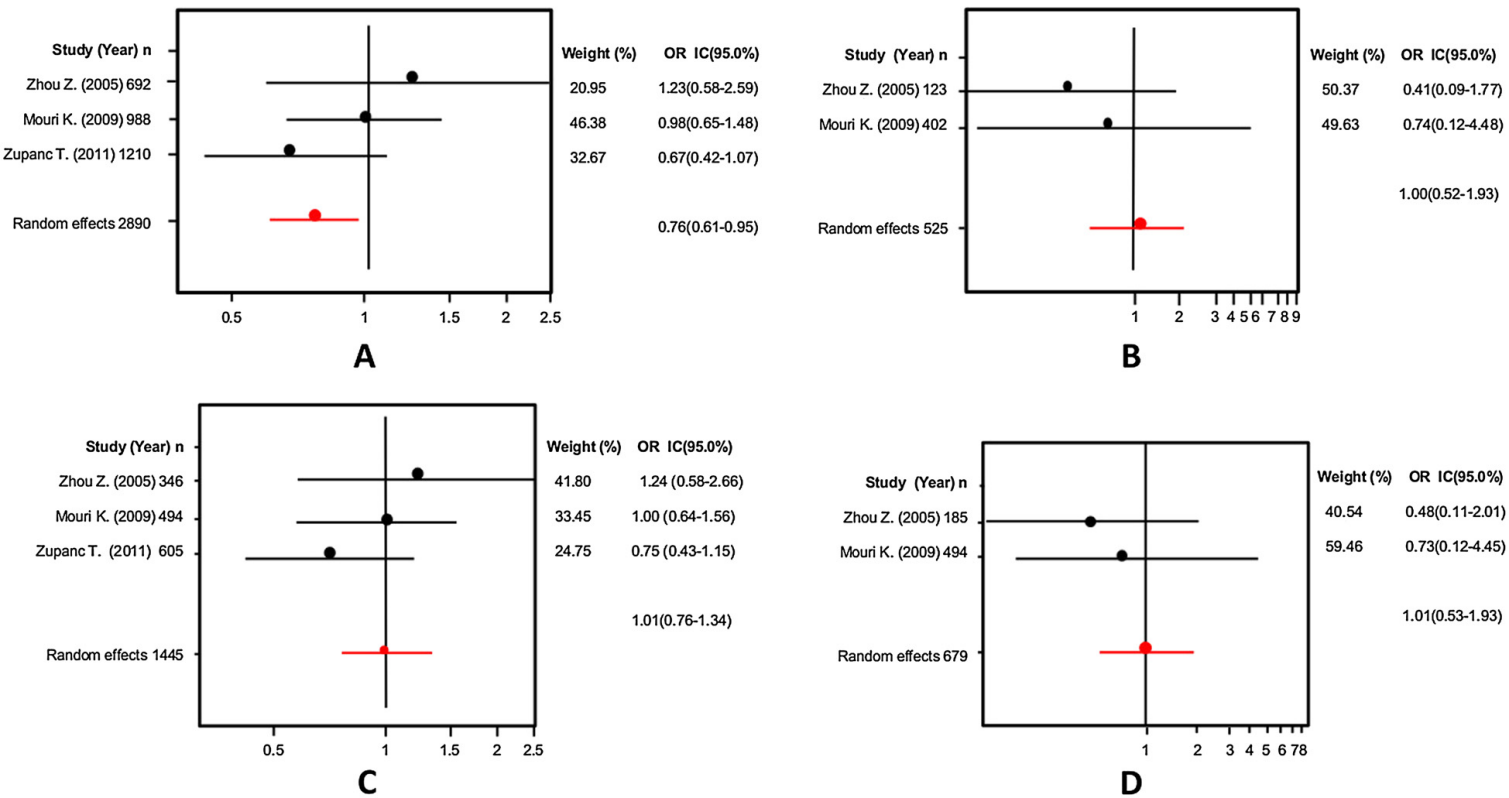
Odds ratios and forest plots of the A-473 T TPH-2 variant in overall studies without heterogeneity using the following models: A) Allelic; B) Additive; C) Dominant, and D) Recessive.

**Figure 10:**
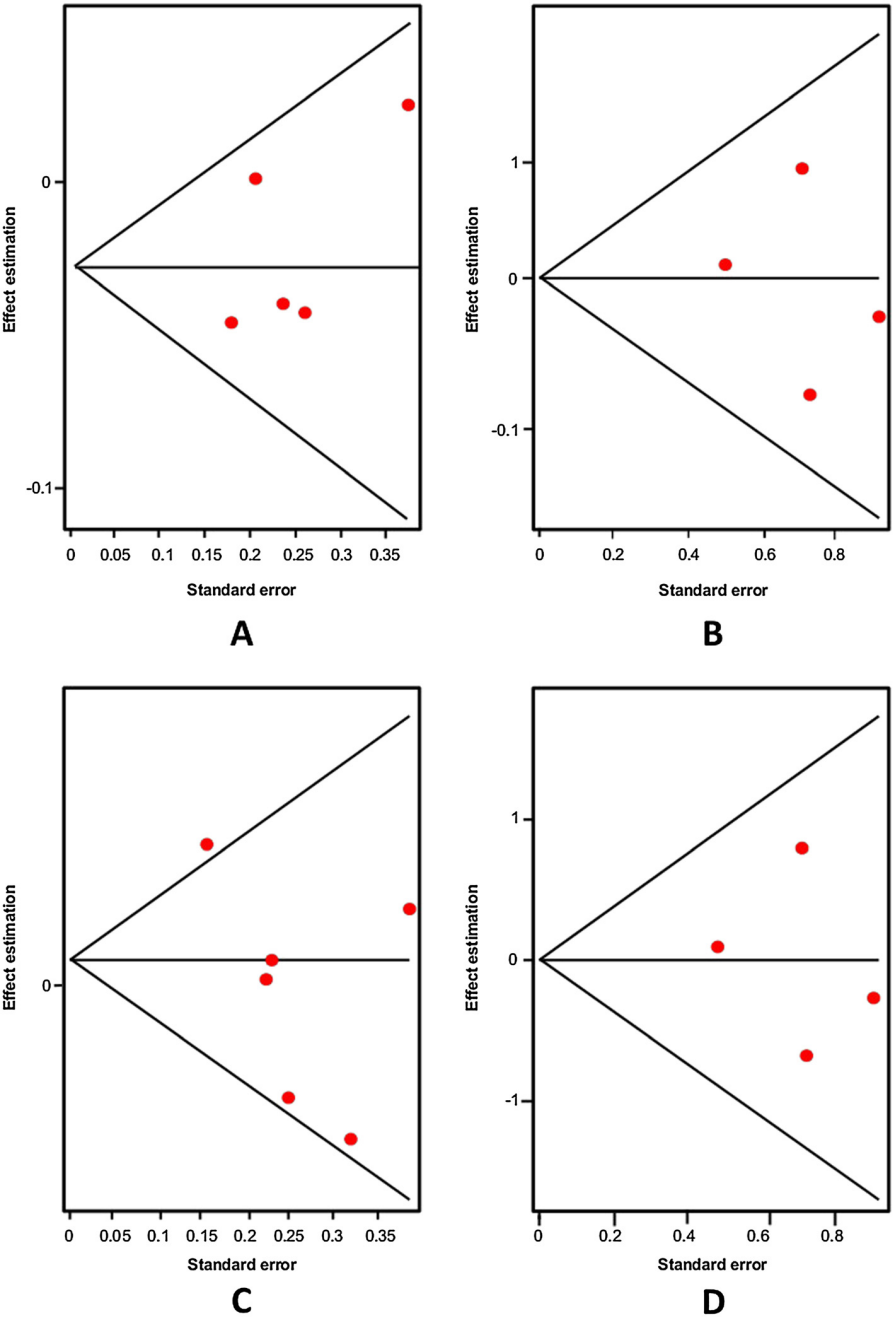
Egger's funnel plots indicating publication bias in studies on suicidal behavior and the A-473 T TPH-2 variant without heterogeneity, using the following models: A) Allelic; B) Additive; C) Dominant, and D) Recessive.

Also, we conducted an analysis of allele A of the A-473 T (rs11178997) polymorphism in association with SB and we found the same negative results with heterogeneity (Random effects: OR: 0.89; 95% CI 0.63-1.26; p(Z) =0.98; Q test = 18.81 df = 5; p = 0.002). No evidence of publication bias was found (Egger’s test t = 0.83, df = 4; p = 0.44). Next, we eliminated heterogeneity by excluding the study by Zhou et al. [61] in a Caucasian population (Random effects: OR:0.76; 95% CI 0.61-0.95; p(Z) =0.80; Q test = 4.54; df = 4; p = 0.33; Figure 9A) and found no significant differences. Besides, we did not encounter evidence of publication bias (Egger’s test: t = 1.04; df = 3; p = 0.37; Figure 10A). When we used the additive model, we excluded the studies by Zupanc et al. [19] and Zhou et al. [61] in the Caucasian population given that some frequencies were equal to zero. Still, we did not find any significant differences without heterogeneity (Random effects: OR: 1.00; 95% CI 0.52-1.93; p(Z) =0.73; Figure 9B; Q test = 2.91; df = 3; p = 0.40); also we did not encounter evidence of publication bias (Egger’s test: t = 0.36, df = 2; p = 0.74; Figure 10B). A negative result was also found for the dominant model without heterogeneity (Random effects: OR: 1.01; 95% CI 0.76-1.34; p(Z) =0.70; Figure 9C; Q test = 10.46; df = 5; p = 0.06); no evidence of publication bias was encountered (Egger’s test: t = 1.74; df = 4; p = 0.15; Figure 10C). The same negative results were obtained with the recessive model without heterogeneity excluding the studies by Zupanc et al. [19] and Zhou et al. [61] in the Caucasian population because some frequencies were equal to zero (Random effects: OR:1.01; 95% CI 0.53-1.93; p(Z) =0.73; Figure 9D; Q test = 2.33; df = 3; p = 0.50); there was no evidence of publication bias (Egger’s test: t = 0.31; df = 2; p = 0.78; Figure 10D). In addition, with regard to the analysis of the A-473 T variant of the TPH-2 gene in relation to SB, we conducted an analysis in the Caucasian population, using the same four models as above and could not find any significant differences (Table 4).

#### G19918A polymorphism

**Figure 11:**
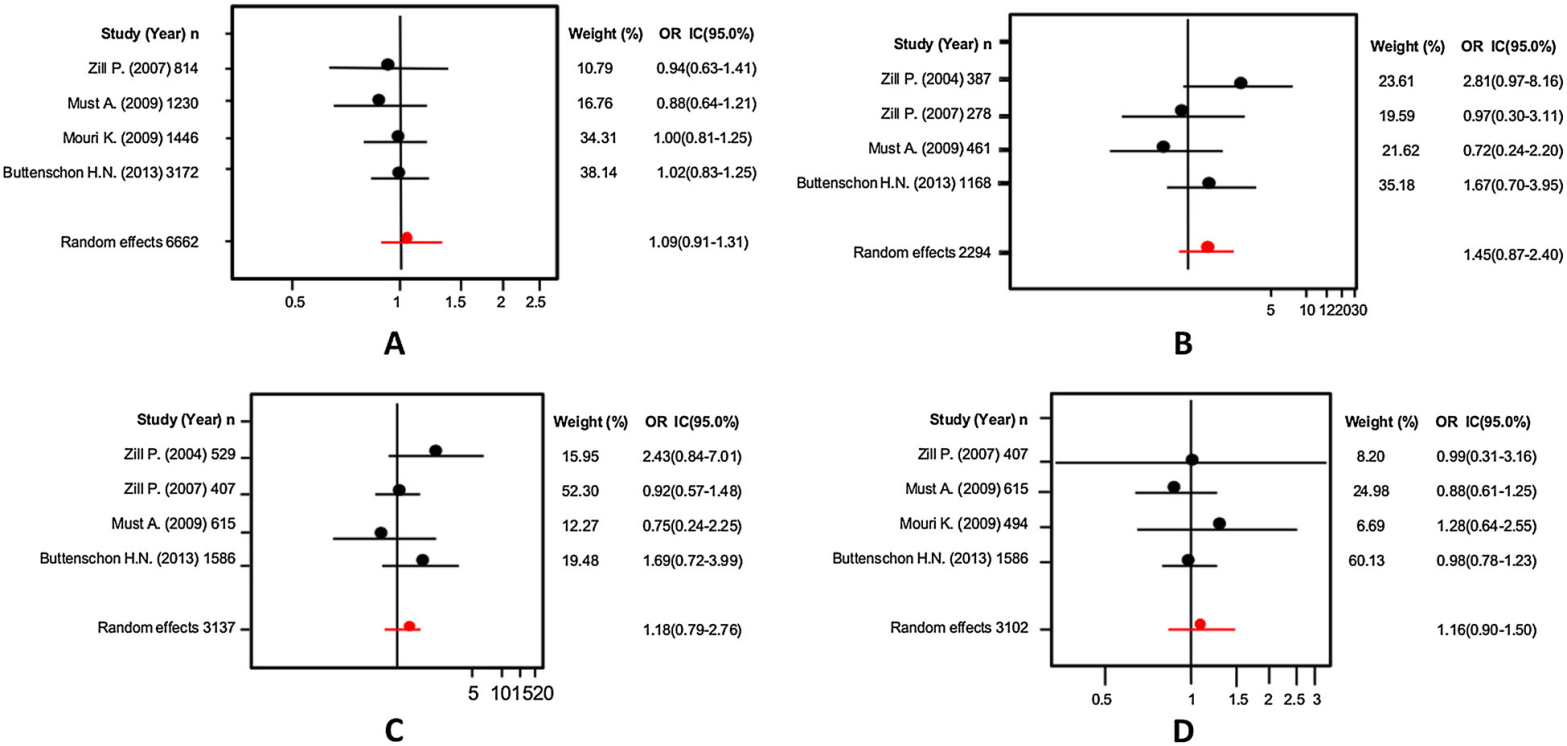
Odds ratios and forest plots of the G19918A TPH-2 variant in overall studies without heterogeneity, using the following models: A) Allelic; B) Additive; C) Dominant, and D) Recessive.

**Figure 12:**
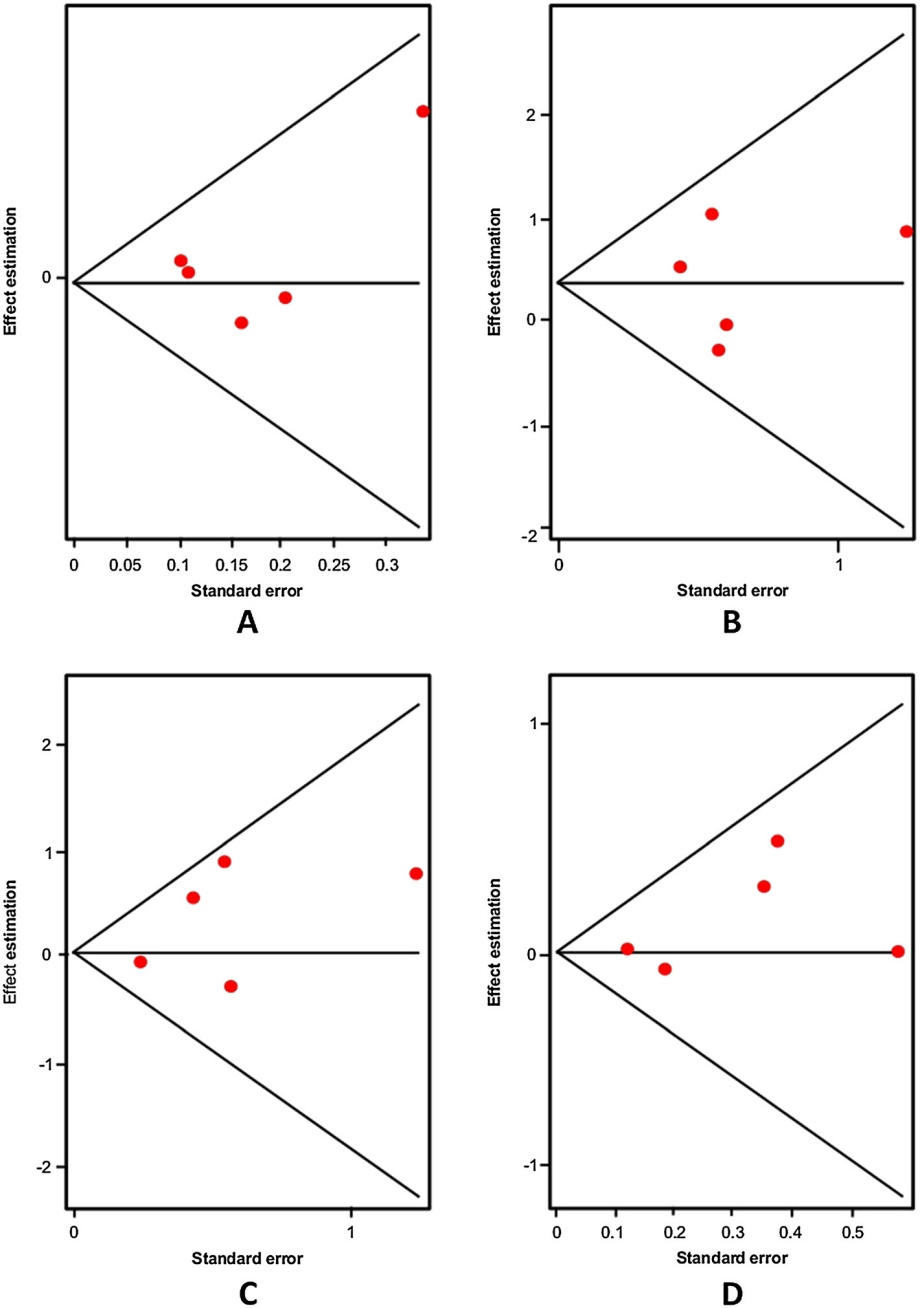
Egger's funnel plots indicating publication bias in studies on suicidal behavior and the G19918A TPH-2 variant without heterogeneity, using the following models: A) Allelic; B) Additive; C) Dominant, and D) Recessive.

Finally, the G19918A (rs1386494) polymorphism was the last variant studied in association with SB. When we analyzed allele G vs allele A, we could not find any significant differences in the analysis without heterogeneity (Random effects: OR: 1.09; 95% CI 0.91-1.31; p(Z) =0.98; Figure 11A; Q test = 10.32; df = 5; p = 0.06); we found no evidence of publication bias (Egger’s test: t = 0.78; df = 4; p = 0.47; Figure 12A). Subsequently, we performed an analysis with the additive model without heterogeneity (Random effects: OR: 1.45; 95% CI 0.87-2.40; p(Z) =0.80; Figure 11B; Q test = 3.70; df = 4; p = 0.44); we found no evidence of publication bias (Egger’s test: t = 0.09; df = 3; p = 0.95, Figure 12B). The same negative outcome was found when using the dominant model without heterogeneity (Random effects: OR: 1.18; 95% CI 0.79-1.76; p(Z) =0.80; Figure 11C; Q test = 4.38; df = 4; p = 0.35); no evidence of publication bias was found (Egger’s test: t = 1.14; df = 3; p = 0.33; Figure 12C). The same result was obtained with the recessive model without heterogeneity (Random effects: OR: 1.16; 95% CI 0.90-1.50; p(Z) =0.45; Figure 11D; Q test = 9.12; df = 5; p = 0.10); no evidence of publication bias was encountered (Egger’s test: t = 0.74; df = 4; p = 0.49; Figure 12D). Finally, we conducted an analysis in the Caucasian population separately using the same four models as in the other polymorphism, and could not find any significant differences (Table 4).

## Discussion

In this study, we explored the association of THP-1 and TPH-2 gene polymorphisms with suicidal behavioral. We found an association between A218C/ATT9C polymorphism of TPH1 and suicidal behavior. However, we could not find an association between TPH2 gene variants and this behavior. To our knowledge, this is the first study evaluating TPH2 gene variants and suicidal behavior.

Suicide has become a serious problem around the world. Various published studies show the search for possible genetic markers that could help in the early detection and prevention of suicidal behavior [13,67,68]. One possible mechanism by which genetic factors may affect the risk for suicide is through the control of serotonergic neurotransmission. TPH genes have been proposed as the main candidate genes in association studies on suicidal behavior, given that the TPH enzyme is a rate-limiting step in serotonin biosynthesis. Despite the increasing amount of genetic association studies on SB, the results are inconclusive. Therefore, we performed a meta-analysis in order to evaluate the association of TPH-1 and TPH-2 genes with SB in which we integrated the outcomes of the different polymorphic markers and different ethnic groups. Initially, although variants A218C (rs1800532) and A779C (rs1799913) of the TPH-1 gene have been studied in relation to suicidal behavior in different meta-analyses [18,22-25], we analyzed the TPH-1 gene in patients with SB. The evaluated variants of this gene were A779C (rs1799913) and A218C (rs1800532); these two polymorphisms are the most studied of the TPH-1 gene and with interesting outcomes. We not only analyzed allele A vs allele C, but also evaluated the association using three other models: additive, dominant and recessive in a pool population that combined the genetic studies of TPH-1 variants. In addition, we performed a sub-group analysis in Caucasian and Asian populations separately to evaluate the association of A779C (rs1799913) and A218C (rs1800532) polymorphisms with SB using the same four models. Our results are in agreement with previous meta-analyses [18,22-25]. These studies report a strong association with SB using the fixed effects model. In the present meta-analysis we also found an association when we used the fixed effects model, but not with the random effects model. However, this result should be taken with caution because the random effects model is more conservative than the fixed effects one. The latter assumes the same true genetic effects, and the former supposes normally distributed effects and parametrizes inter-studies variations. To get a clearer panorama of the possible role of these variants in SB, we summarized the results of our analyses in Tables 3 and 4. The evidence suggests that TPH-1 A218C/A779C polymorphisms may be associated with increased susceptibility to present SB clinically.

On the other hand, to explore the association of SB with TPH-2 gene, we chose rs4570625 (G-703 T), rs11178997 (A-473 T) and rs1386494 (G19918A) variants, given their functionality and the extensive amount of studies evaluating these polymorphisms in association with SB. We performed the analysis with the same four models used for the TPH1-gene: allelic, additive, dominant, and recessive. We also conducted a sub-group analysis in the Caucasian and Asian populations utilizing the same previously mentioned models. In the present study we could not find any association in any of the models, but we found a slight protective effect concerning polymorphism A-473 T toward suicidal behavior in the recessive model (Random effects: OR: 0.76; 95% CI 0.61-0.95; p(Z) =0.80; Q test = 4.54; df = 4; p = 0.33); no evidence of publication bias was encountered (Egger’s test: t = 1.04; df = 3; p = 0.37).

There are several explanations for the present outcomes concerning the lack of association of TPH-2 variants with SB. First, the criteria used in the selection of the control groups differ among studies. Second, although the subjects were patients with SB, there were differences in the diagnoses (attempted, ideation and completed suicide, and exclusion criteria of neuropsychiatric diseases, among other factors). For example, Souery et al. [37] work with patients with bipolar disorder that present SB, Yoon and Kim [44] with depressive subjects and SB, Paik et al. [45] with schizophrenic patients, and Turecki et al. [39] with suicide completers only. Other possibility is genetic heterogeneity: some authors have shown that the distribution of the frequencies of TPH-2 variants may depend on ethnicity. Therefore, we evaluated the association of TPH2 variants with SB in several populations. The fourth explanation considers the presence of other genes which may interact with TPH-2 to increase the risk to present SB clinically. We also want to emphasize the relevance of employing larger sample sizes to enable the observation of more conclusive results.

This study has some limitations. First, we performed the present meta-analysis based only on published studies. We considered that by selecting only published results, we ensured that our meta-analysis excluded poorly designed studies. Second, although the present analysis involves a large number of studies and genetic variants, it is relatively small in comparison with other meta-analysis on different diseases. Also, we could not perform an analysis including suicide ideation, suicide attempt, or completed suicide. Other limitation is that we did not stratify the analysis by gender because it was not always possible to obtain all the data from the reports or directly from the authors. Also, we did not perform the analysis of all the variants of the TPH-2 gene in the Asian population because there were not enough studies. Sixth, we did not evaluate SB endophenotypes or psychopathologies which could increase the risk for suicidal behavior. One last comment: is that although there are previous meta-analyses evaluating the association of the TPH-1 gene (A218C and A779C variants) and SB, our study contains more recent data and the use of various types of models to perform the analysis.

## Conclusion

In summary, we performed a meta-analysis to assess the association of A779C (rs1799913) and A218C (rs1800532) variants of the TPH-1 gene, and rs4570625 (G-703 T), rs11178997 (A-473 T), and rs1386494 (G19918A) of the TPH-2 gene with SB. Our analysis of TPH-1 provided evidence that A218C/A779C TPH-1 variants may be risk factors to present SB, which is in agreement with previously reported meta-analyses [18,22-25]. With regard to G-703 T/A-473 T/G19918A TPH-2 variants, our updated meta-analysis did not detect any significant association with patients presenting SB. However, these results should be interpreted with caution because not all the models detected this association and the sample size may be just enough to detect small effects of the polymorphisms. Further studies with larger samples in different ethnic populations need to be undertaken to confirm our findings.

## Competing interests

The authors declare that they have no competing interests.

## Authors’ contributions

TZC and GCTB conceived the study, participated in its design and helped to draft the manuscript. TZC, JRI, and LNML helped to perform the statistical analysis and to draft the manuscript. All authors read and approved the final manuscript.

## Pre-publication history

The pre-publication history for this paper can be accessed here:

http://www.biomedcentral.com/1471-244X/14/196/prepub

## Supplementary Material

Additional file 1: Table S1 Summary finding of studies association between TPH1 gene variants and suicidal behavior. **Table S2.** Methodological quality of TPH1 gene variants studies association included based on the Newcastle-Ottawa scale. Click here for file
