# Supplementary material for: Association of TPH-1 and TPH-2 gene polymorphisms with suicidal behavior: a systematic review and meta-analysis
Source: BMC Psychiatry. 2014 Jul 8;14:196. doi: 10.1186/1471-244X-14-196 (PMC4099217; doi:10.1186/1471-244X-14-196)
Supplement: Additional file 1: Table S1 — Summary finding of studies association between TPH1 gene variants and suicidal behavior. Table S2. Methodological quality of TPH1 gene variants studies association included based on the Newcastle-Ottawa scale. [file 1471-244X-14-196-S1.docx]

**Additional file 1: Table S1. Summary finding of studies association between TPH1 gene variants and suicidal behavior.**

| **Reference** | **OR (CI 95%)** | **Number of patients (studies)** | **Design** | **Quality evidence (GRADE)** | **Publication bias** |
| --- | --- | --- | --- | --- | --- |
| Bellivier F. et al., 1998 | 2.27(1.51-3.41) | 104 | Case-control | ⊕⊕⊕○  Moderate | Undetected |
| Geijer T.et al., 2000 | 1.89(1.22-2.92) | 165 | Case-control | ⊕⊕⊕○  Moderate | Undetected |
| Du L. et al., 2000 | 1.22(0.69-2.13) | 35 | Case-control | ⊕⊕⊕⊕  High | Undetected |
| Souery D. et al., 2001 | 1.20(0.88-1.64) | 167 | Case-control | ⊕⊕⊕○  Moderate | Undetected |
| Abbar M. et al., 2001 | 1.33(0.98-1.79) | 231 | Case-control | ⊕⊕⊕○  Moderate | Undetected |
| Zalsman G.et al., 2001 | 0.90(0.56-1.43) | 84 | Case-control | ⊕⊕⊕○  Moderate | Undetected |
| Turecki G. et al., 2001 | 1.00(0.69-1.45) | 101 | Case-control | ⊕⊕⊕⊕  High | Undetected |
| Rujescu D. et al., 2002 | 1.36(0.81-2.29) | 86 | Case-control | ⊕⊕⊕○  Moderate | Undetected |
| Rujescu D. et al., 2003 | 1.28(0.88-1.86) | 147 | Case-control | ⊕⊕⊕⊕  High | Undetected |
| Pooley E.C. et al., 2003 | 1.04(0.71-1.51) | 129 | Case-control | ⊕⊕⊕○  Moderate | Undetected |
| Jernej B. et al., 2004 | 1.05(0.80-1.37) | 192 | Case-control | ⊕⊕⊕○  Moderate | Undetected |
| Stefulj J. et al., 2005 | 1.00(0.76-1.31) | 160 | Case-control | ⊕⊕⊕⊕  High | Undetected |
| Stefulj J. et al., 2006 | 1.00(0.79-1.26) | 247 | Case-control | ⊕⊕⊕○  Moderate | Undetected |
| Viana M.M. et al., 2006 | 0.99(0.63-1.57) | 248 | Case-control | ⊕⊕⊕○  Moderate | Undetected |
| Baud P. et al., 2009 | 1.04(0.90-1.21) | 537 | Case-control | ⊕⊕⊕○  Moderate | Undetected |
| Wilson T.et al., 2009 | 1.28(0.83-1.97) | 71 | Case-control | ⊕⊕⊕○  Moderate | Undetected |
| Saetre P. et al., 2010 | 1.61(1.35-1.92) | 299 | Case-control | ⊕⊕⊕○  Moderate | Undetected |
| Buttenschon H.N. et al., 2013 | 1.74(1.44-2.10) | 490 | Case-control | ⊕⊕⊕⊕  High | Undetected |
| Tsai S.J. et al., 1999 | 1.83(1.13-2.97) | 41 | Case-control | ⊕⊕⊕○  Moderate | Undetected |
| Liu D. et al., 2006 | 0.92(0.68-1.26) | 287 | Case-control | ⊕⊕⊕○  Moderate | Undetected |
| Yoon H.K. et al., 2008 | 1.05(0.71-1.54) | 193 | Case-control | ⊕⊕⊕○  Moderate | Undetected |
| Kunugi H. et al.,1999 | 1.06(0.67-1.67) | 46 | Case-control | ⊕⊕⊕○  Moderate | Undetected |
| Paik I. et al., 2000 | 0.53(0.29-0.95) | 27 | Case-control | ⊕⊕⊕○  Moderate | Undetected |
| Ono H., et al., 2000 | 1.00(0.68-1.46) | 132 | Case-control | ⊕⊕⊕○  Moderate | Undetected |
| Hong C.J. et al., 2001 | 1.31(0.97-1.75) | 140 | Case-control | ⊕⊕⊕⊕  High | Undetected |
| Ohtani M. et al., 2004 | 0.74(0.53-1.02) | 134 | Case-control | ⊕⊕⊕○  Moderate | Undetected |
| Nielsen D.A. et al.,1998 | 1.02(0.73-1.42) | 102 | Case-control | ⊕⊕⊕○  Moderate | Undetected |
| Rotondo A. et al., 1999 | 0.57(0.40-0.81) | 97 | Case-control | ⊕⊕⊕⊕  High | Undetected |
| Roy A. et al., 2001 | 0.51(0.27-0.97) | 24 | Case-control | ⊕⊕⊕○  Moderate | Undetected |

**Table S2. Methodological quality of TPH1 gene variants studies association included based on the Newcastle-Ottawa scale**

| **Reference** | **Selection** | **Comparability** | **Exposure** | **Total score** |
| --- | --- | --- | --- | --- |
| Bellivier F. et al., 1998 |  |  |  | 8 |
| Geijer T.et al., 2000 |  |  |  | 7 |
| Du L. et al., 2000 |  |  |  | 8 |
| Souery D. et al., 2001 |  |  |  | 6 |
| Abbar M. et al., 2001 |  |  |  | 7 |
| Zalsman G.et al., 2001 |  |  |  | 7 |
| Turecki G. et al., 2001 |  |  |  | 8 |
| Rujescu D. et al., 2002 |  |  |  | 7 |
| Rujescu D. et al., 2003 |  |  |  | 6 |
| Pooley E.C. et al., 2003 |  |  |  | 6 |
| Jernej B. et al., 2004 |  |  |  | 7 |
| Stefulj J. et al., 2005 |  |  |  | 7 |
| Stefulj J. et al., 2006 |  |  |  | 7 |
| Viana M.M. et al., 2006 |  |  |  | 7 |
| Baud P. et al., 2009 |  |  |  | 7 |
| Wilson T.et al., 2009 |  |  |  | 7 |
| Saetre P. et al., 2010 |  |  |  | 6 |
| Buttenschon H.N. et al., 2013 |  |  |  | 8 |
| Tsai S.J. et al., 1999 |  |  |  | 7 |
| Liu D. et al., 2006 |  |  |  | 7 |
| Yoon H.K. et al., 2008 |  |  |  | 7 |
| Kunugi H. et al.,1999 |  |  |  | 7 |
| Paik I. et al., 2000 |  |  |  | 7 |
| Ono H., et al., 2000 |  |  |  | 6 |
| Hong C.J. et al., 2001 |  |  |  | 6 |
| Ohtani M. et al., 2004 |  |  |  | 7 |
| Nielsen D.A. et al.,1998 |  |  | c | 7 |
| Rotondo A. et al., 1999 |  |  |  | 6 |
| Roy A. et al., 2001 |  |  |  | 6 |
